# ﻿Two new species of *Aphis* (*Toxoptera*) Koch (Hemiptera, Aphididae) from China

**DOI:** 10.3897/zookeys.1172.106518

**Published:** 2023-07-24

**Authors:** Zhentao Cheng, Xiaolei Huang

**Affiliations:** 1 State Key Laboratory of Ecological Pest Control for Fujian and Taiwan Crops, College of Plant Protection, Fujian Agriculture and Forestry University, Fuzhou 350002, China Fujian Agriculture and Forestry University Fuzhou China

**Keywords:** Aphids, *COI*, DNA barcode, identification key, morphology, taxonomy

## Abstract

Two new aphid species, Aphis (Toxoptera) fafuensis Cheng & Huang, **sp. nov.**, feeding on *Adinandramillettii* (Pentaphylacaceae) from Fujian, China, and Aphis (Toxoptera) sennae Cheng & Huang, **sp. nov.**, feeding on *Sennabicapsularis* (Fabaceae) from Yunnan, China, were described. Morphological characters and molecular data supported the taxonomic position of the new species within the subgenus Aphis (Toxoptera). A key for identifying species of apterous viviparous females in this subgenus is provided.

## ﻿Introduction

The aphid genus *Toxoptera* was first proposed by Koch (1856), with *T.aurantiae* Koch, 1856 designated as the type species. However, the name was considered as a synonym and the type species was revised as *T.aurantii* Boyer de Fonscolombe, 1841 by Schouteden (1906). Baker (1920) defined *Toxoptera* as having alate with once-branched media of the forewing. Williams (1921) described another unique character of this genus, which is a stridulatory apparatus consisting of peg-like spines on the hind tibiae and ventro-lateral spinulose ridges on the posterior abdominal segments. After Börner (1930) erected the genus *Schizaphis* to include species characterized by the once-branched media of the forewing, *Toxoptera* had been distinguished from allied genera by the presence of the stridulatory apparatus.

Kim and Lee (2008) investigated the phylogenetic relationships within the tribe Aphidini using several gene markers including *tRNA/COII*, *12S/16S* and *EF1-α*, and their results showed that *Toxoptera* may be non-monophyletic. Based on *COI* sequences, Wang and Qiao (2009) showed that *T.odinae* was phylogenetically distinct from other *Toxoptera* species and should be reverted to Aphis (Aphis) odinae van der Goot, 1917 (Blackman et al. 2011). Then a molecular phylogenetic study of *Aphis* species based on nuclear and mitochondrial genes confirmed that Toxoptera should be regarded as a subgenus of Aphis (Lagos et al. 2014).

Thus far, the subgenus Aphis (Toxoptera) has been represented by five species (Remaudière and Remaudière 1997; Favret 2023): A. (T.) aurantii Boyer de Fonscolombe, 1841, A. (T.) celtis Shinji, 1922, A. (T.) citricidus Kirkaldy, 1907, A. (T.) victoriae Martin, 1991 and A. (T.) chaetosiphon Qiao, Wang & Zhang, 2008. However, it should be noted that A. (T.) celtis is considered a possible synonym of A. (T.) aurantii (Martin 1991; Blackman and Eastop 2023). The host plants for species of this subgenus are very diverse, including Theaceae, Rutaceae, Rubiaceae, and many other plant families. New host plants can provide novel ecological niches for herbivorous insects, contributing to different host preferences and genetic isolation. Therefore, host plant is key to the diversification of herbivorous insects and plays an important role in their speciation (Mitter et al. 1991; Futuyma and Agrawal 2009). Based on multiple gene fragments and the haplotype network analysis, Li et al. (2021) found that the population of A. (T.) aurantii feeding on *Ficus* showed great genetic difference from those feeding on other host plant groups, indicating that A. (T.) aurantii has been undergoing the evolution of host specialization on *Ficus*.

In recent years, while collecting aphid samples in southern China, we obtained some samples that may represent two undescribed species from *Adinandramillettii* (Hook. & Arn.) Benth. & Hook. f. ex Hance (Pentaphylacaceae) and *Sennabicapsularis* (L.) Roxb. (Fabaceae), respectively. By integrating morphological and molecular data, this paper describes the new species and confirms their taxonomic positions within Aphis (Toxoptera).

## ﻿Material and methods

### ﻿Field sampling

The specimens of A. (T.) fafuensis Cheng & Huang, sp. nov. were collected in Fujian, China on *Adinandramillettii* and the samples of A. (T.) sennae Cheng & Huang, sp. nov. were collected in Yunnan, China on *Sennabicapsularis*. The detailed collection information is provided in Suppl. material 1: table S1. All samples were preserved in 95% ethanol and kept at -80 °C for further morphological measurement and molecular experiments.

### ﻿Morphological description

Six apterous viviparous females of A. (T.) fafuensis Cheng & Huang, sp. nov. and eight apterous viviparous females of A. (T.) sennae Cheng & Huang, sp. nov. were slide-mounted in Canada Balsam. Aphid terminology and the morphological measurements used in this paper followed Qiao et al. (2008) (Table 1). All specimens were examined and measurements and images were taken by using Nikon SMZ18 stereomicroscope. The measurements and the micrographs of mounted specimens were performed using a computer-connected Nikon set: Nikon Eclipse Ci-L upright microscope, 16MP digital camera with 0.55X adapter and imaging software NIS-Elements D ver. 4.60.00. The unit of measurement in this paper is millimeters (mm).

**Table 1.** Biometric data (mean, range) of Aphis (Toxoptera) fafuensis Cheng & Huang, sp. nov. and Aphis (Toxoptera) sennae Cheng & Huang, sp. nov. (in mm).

Parts		A. (T.) fafuensis Cheng & Huang, sp. nov. Apterous vivipara (*N* = 6)		A. (T.) sennae Cheng & Huang, sp. nov. Apterous vivipara (*N* = 8)	
		mean	range	mean	range
Length (mm)	BL	1.11	0.91–1.19	1.64	1.50–1.89
	BW	0.73	0.66–0.77	1.20	1.02–1.40
	URS	0.10	0.09–0.11	0.12	0.11–0.13
	WA	1.06	0.83–1.16	1.35	1.34–1.38
	Ant. I	0.06	0.06–0.07	0.08	0.07–0.08
	Ant. II	0.06	0.05–0.06	0.07	0.06–0.07
	Ant. III	0.25	0.18–0.28	0.33	0.31–0.36
	Ant. III_WD	0.02	0.02–0.02	0.03	0.03
	Ant. IV	0.17	0.13–0.19	0.21	0.20–0.23
	Ant. V	0.17	0.13–0.20	0.22	0.21–0.22
	Ant. VIb	0.07	0.05–0.08	0.09	0.09–0.10
	PT	0.29	0.23–0.31	0.35	0.34–0.37
	HF	0.35	0.29–0.38	0.48	0.44–0.53
	HF_WD	0.06	0.05–0.06	0.08	0.07–0.09
	HT	0.62	0.52–0.70	0.90	0.83–1.00
	HT_WD	0.03	0.03–0.04	0.05	0.05
	2HT	0.07	0.06–0.07	0.10	0.09–0.11
	SIPH	0.15	0.12–0.17	0.19	0.17–0.20
	SIPH_BW	0.07	0.05–0.08	0.08	0.07–0.10
	SIPH_DW	0.04	0.03–0.04	0.04	0.04
	Cauda	0.14	0.12–0.15	0.19	0.17–0.20
	Cauda_BW	0.08	0.06–0.10	0.13	0.11–0.15
	Hairs on Ant. III	0.01	0.01	0.01	0.01
	Hairs on HF	0.02	0.01–0.02	0.02	0.02
	Hairs on HT	0.02	0.02–0.03	0.03	0.02–0.03
No. of hairs on	URS		6		6
	Ant. I		5–6		4
	Ant. II		3–4		4
	Ant. III		4–8		5–9
	Ant. IV		3–5		3–5
	Ant. V		2–4		3–4
	Ant. VIb		3		2–3
	PT		5–6		4–6
	HF		21–36		23–35
	HT		58–69		82–95
	Cauda		14–21		9–17
	AP		18–21		20–29
	GP		11–18		8–13
	Gonapophyses		9–13		12–16
Ratio (times)	BL/BW	1.5	1.4–1.6	1.4	1.3–1.5
	WA/BL	1.0	0.8–1.0	0.9	0.8–0.9
	HT/BL	0.6	0.5–0.6	0.6	0.5–0.6
	HF/BL	0.3	0.3	0.3	0.3
	SIPH/BL	0.1	0.1	0.1	0.1
	PT/WA	0.3	0.3	0.3	0.3
	Ant. III/WA	0.2	0.2	0.2	0.2–0.3
Ratio (times)	PT/Ant. VIb	4.2	3.8–4.6	3.8	3.4–4.1
	URS/URS_BW	2.5	2.2–2.8	2.6	2.0–3.3
	URS/2HT	1.6	1.4–1.8	1.3	1.2–1.4
	SIPH/Cauda	1.0	0.9–1.2	1.0	0.9–1.1
	Cauda_BW/Cauda	0.5	0.4–0.7	0.7	0.6–0.8
	HF/Ant. III	1.5	1.3–1.6	1.4	1.4–1.5
	2HT/Ant. III	0.3	0.3	6.1	5.7–6.7
	URS/Ant. III	0.4	0.4–0.5	0.4	0.3–0.4
	Ant. III_H/Ant. III_WD	0.5	0.5	0.3	0.3
	HT_H/Ant. III_WD	1.2	1.0–1.5	0.8	0.8–1.0
	SIPH/Ant. III_WD	7.3	6.0–8.5	0.3	0.3
	Ant. III_WD/SIPH_BW	0.3	0.3–0.4	0.4	0.3–0.4

The following abbreviations have been used: BL, body length; BW, body width; URS, ultimate rostral segment; URS_BW, basal width of URS; WR, whole length of rostral; WA, whole length of antenna; Ant. I, Ant. II, Ant. III, Ant. IV, Ant. V, Ant. VIb, for antennal segments I, II, III, IV, V and the base of Ant. IV, respectively; Ant. III_WD, the widest diameter of Ant. III; PT, processus terminalis; PT_WD, the widest diameter of PT; HF, hind femur; HF_WD, the widest diameter of HF; HT, hind tibia; HT_WD, the widest diameter of HT; 2HT, second hind tarsal segment; SIPH, siphunculus; SIPH_BW, basal width of siphunculus; SIPH_DW, distal width of siphunculus; Cauda_BW, basal width of cauda; AP, anal plate; GP, genital plate; gona, gonapophyses.

To examine the possible morphological differences between the two newly discovered species and A. (T.) aurantii, a one-way analysis of variance (ANOVA) was conducted. Furthermore, to identify pairwise differences of the morphological characters of specimens, post hoc multiple comparisons were performed using the Least Significant Difference (LSD) test (Suppl. material 1: table S2). All statistical analyses were carried out using SPSS ver. 24 (IBM, Chicago, IL, USA).

### ﻿Molecular analysis

The whole genomic DNA of each sample was extracted from the single individual preserved in 95% ethanol using the DNeasy Blood & Tissue Kit (Qiagen, Hilden, Germany). The standard DNA barcode gene of aphids, cytochrome c oxidase subunit I (COI) was amplified with primer LepF (5’-ATTCAACCAATCATAAAGATATTGG-3’) and LepR (5’-TAAACTTCTGGATGTCCAAAAAATCA-3’) (Foottit et al. 2008). PCR amplifications were performed in a final volume of 25 µL reaction mixture containing 2 μL of template DNA, 0.5 μL of both forward and reverse primer (10 μM), 0.25 μL of Taq DNA polymerase (5 U/μL), 17.25 μL of double distilled H_2_O, 2.5 μL of 10× buffer and 2 μL of dNTP. PCR thermal regime was as follows: 5 min of initial denaturation at 95 °C, 35 cycles of 20 s at 94 °C, 30 s at 50 °C (the annealing temperature) and 2 min at 72 °C, and 10 min of final extension at 72 °C. The products of PCR were visualized by electrophoresis on a 1% agarose gel and then bidirectionally sequenced at Beijing Tsingke Biotech Co., Ltd. (Beijing, China).

The maximum-likelihood phylogenetic tree base on *COI* sequences includes thirty-six samples representing the two new species and three Aphis (Toxoptera) species including A. (T.) aurantii, A. (T.) citricidus and A. (T.) chaetosiphon were reconstructed. A. (T.) celtis and A. (T.) victoriae were excluded from the phylogenetic analysis, as A. (T.) celtis did not have any available *COI* sequence and the sequences of A. (T.) victoriae in GenBank did not provide enough sites for analysis after alignment with other sequences. Aphis (Aphis) gossypii Glover, 1877 and Aphis (Aphis) odinae were used as outgroups (Fig. 3, Table 2). All sequences were assembled by ContigExpress (Vector NTI Suite 6.0, InforMax Inc.), and the reliability was checked by BLAST. Multiple alignment was conducted using MAFFT (Katoh and Standley 2013) based on the default setting. Maximum-likelihood phylogenies were inferred using PhyloSuite ver. 1.2.3 (Zhang et al. 2020) under the TIM2+I+F model for 5000 ultrafast bootstraps. The mean genetic distances among the seven *Aphis* species used for phylogenetic analysis were calculated using MEGA 7 (Kumar et al. 2016) under Kimura’s two-parameter (K2P) model (Kimura 1980).

**Table 2.** Voucher information and GenBank accession numbers of aphid samples used in molecular data analysis.

Species	Voucher number	Host plant	Location	Accession Number
A. (T.) aurantii	HL_20150518_4	*Ilexlatifolia*	Fuzhou, Fujian	MH821442
A. (T.) aurantii	HL_20150530_3	*Micheliaalba*	Fuzhou, Fujian	OK285285
A. (T.) aurantii	HL_20150705_2	*Camelliasinensis*	Fuzhou, Fujian	OQ985354
A. (T.) aurantii	HL_20150705_3	*Camelliasinensis*	Fuzhou, Fujian	OQ985355
A. (T.) aurantii	HL_20150907_1	*Loropetalumchinense*	Fuzhou, Fujian	MH821475
A. (T.) aurantii	HL_20150907_2	*Ilexcornuta*	Fuzhou, Fujian	MH821486
A. (T.) aurantii	HL_20160119_1	*Citrusmaxima*	Fuzhou, Fujian	MH821519
A. (T.) aurantii	HL_20160212_1	*Pittosporumtobira*	Changsha, Hunan	OQ985356
A. (T.) aurantii	HL_20160409_6	*Ficuselastica*	Fuzhou, Fujian	OQ985357
A. (T.) aurantii	HL_20160412_13	*Scheffleraactinophylla*	Fuzhou, Fujian	MH821564
A. (T.) aurantii	HL_20160607_2	*Adinandramillettii*	Fuzhou, Fujian	MH821575
A. (T.) aurantii	HL_20161118_1	*Camelliasinensis*	Fuzhou, Fujian	MH821619
A. (T.) aurantii	HL_20170429_29	*Gleditsiasinensis*	Hangzhou, Zhejiang	OQ985358
A. (T.) aurantii	HL_20170429_35	*Camelliacuspidata*	Hangzhou, Zhejiang	MH821131
A. (T.) aurantii	HL_20170521_3	*Camellia* sp.	Fuzhou, Fujian	MH821175
A. (T.) aurantii	HL_20170609_19	*Schimasuperba*	Fuzhou, Fujian	MH821220
A. (T.) aurantii	HL_20170614_13	*Murrayaexotica*	Fuzhou, Fujian	MH821231
A. (T.) aurantii	HL_20170811_8	*Citrusreticulata*	Xishuangbanna, Yunnan	MH821297
A. (T.) aurantii	HL_20170922_13	*Camelliasinensis*	Fuzhou, Fujian	MH821386
A. (T.) sennae Cheng & Huang, sp. nov.	HL_zld20171111_7	*Sennabicapsularis*	Kunming, Yunnan	OQ985359
A. (T.) fafuensis Cheng & Huang, sp. nov.	HL_20160627_3	*Adinandramillettii*	Fuzhou, Fujian	OQ985360
A. (T.) fafuensis Cheng & Huang, sp. nov.	HL_20150517_5	*Adinandramillettii*	Fuzhou, Fujian	OQ985361
A. (T.) fafuensis Cheng & Huang, sp. nov.	HL_20180423_6	*Adinandramillettii*	Fuzhou, Fujian	OQ985362
A. (T.) chaetosiphon	HL_20180119_1	*Camellia* sp.	Fuzhou, Fujian	ON754448
A. (T.) chaetosiphon	HL_20180423_5	*Camelliaoleifera*	Fuzhou, Fujian	ON754765
A. (T.) chaetosiphon	HL_20150418_7	*Camelliajaponica*	Fuzhou, Fujian	MH821874
A. (T.) chaetosiphon	HL_20151226_7	*Camelliaoleifera*	Fuzhou, Fujian	MH821863
A. (T.) citricidus	HL_20150802_8	*Pyracanthafortuneana*	Xian, Shanxi	MH821930
A. (T.) citricidus	HL_20150821_1	*Citrusreticulata*	Emeishan, Sichuan	MH821941
A. (T.) citricidus	HL_20150907_10	*Citrusreticulata*	Fuzhou, Fujian	MH821952
A. (T.) citricidus	HL_20170205_4	Unknown	Shenzhen, Guangdong	MH821886
A. (T.) citricidus	HL_20180128_4	*Zanthoxylumpiperitum*	Haikou, Hainan	ON754472
A. (T.) citricidus	HL_20180616_8	*Macluratricuspidata*	Hangzhou, Zhejiang	ON754827
A. (T.) citricidus	HL_zld20171101_14	*Citrusreticulata*	Chongzuo, Guangxi	MH821963
A. (A.) odinae	HL_20161017_4	*Rhuschinensis*	Fuzhou, Fujian	MH821355
A. (A.) gossypii	HL_20150822_8	*Salviasplendens*	Leshan, Sichuan	MH821146

### ﻿Specimen deposition

The holotypes and paratypes of the new species and all the other specimens examined here are deposited in the Insect Systematics and Diversity Lab, Fujian Agriculture and Forestry University, Fuzhou, China.

## ﻿Taxonomy

### ﻿ Aphis (Toxoptera) fafuensis

Taxon classificationAnimaliaHemipteraAphididae

Cheng & Huang sp. nov.

340608E9-F6AF-5482-AAEC-05C2EAFEDA6C

https://zoobank.org/13AFFC64-CF53-4E58-8953-6FA9067D43A5

Figs 1 , 4A–C

#### Description.

Apterous viviparous females: Body elliptical (Fig. 4A), dark brown in life, head is slightly lighter in color and the tibiae are markedly pale (Fig. 4B).

**Mounted specimens: *Head*.** Vertex convex, antennal tubercles slightly developed. Head with one pair of cephalic hairs, one pair of antennal tubercular hairs. Dorsum of head smooth with 4–7 hairs. Dorsal hairs of head fine, and with developed small tubercles at bases. Antennae six-segmented, segments I and II dark brown, segments III–VIb and PT dark at distal end and with spinulose imbrications; 0.8–1.0 times as long as body. Length in proportion of segments I–VI: 21–33, 21–28, 100, 61–76, 65–72, 26–33 + 107–138. Processus terminalis 3.8–4.6 times as long as basal part of the segment. Antennal hairs acute, segments I–VI each with 5–6, 3–4, 4–8, 3–5, 2–4, 3 + 5–6 hairs, respectively, apical part of processus terminalis with 0–4 hairs. Length of hairs on segment III 0.01 mm, 0.5 times as long as the widest diameter of segment III. Rostrum long, apical part dark brown, reaching hind coxae or abdominal segment I. Ultimate rostral segment wedge-shaped, 2.2–2.8 times as long as basal width, 1.4–1.8 times as long as second hind tarsal segment. Ultimate rostral segment with four pairs of hairs, including one pair of accessory hairs.

**Figure 1.**
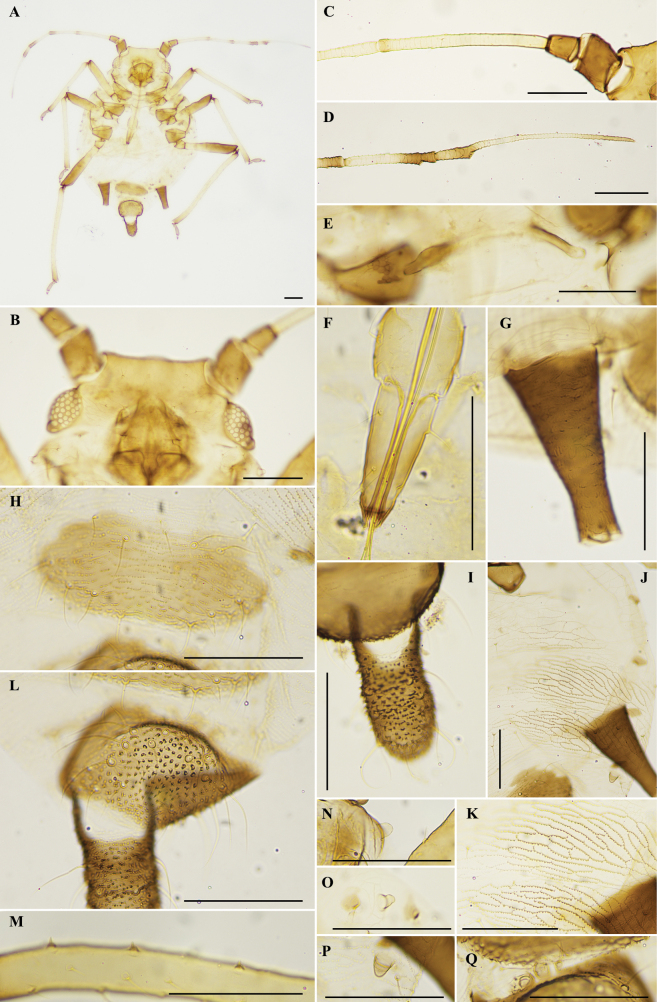
Aphis (Toxoptera) fafuensis Cheng & Huang, sp. nov., apterous viviparous female **A** dorsal view of body **B** dorsal view of head **C** antennal segments I–III **D** antennal segments V–VI **E** mesosternal furca **F** ultimate rostral segment **G** siphunculus **H** genital plate **I** cauda **J** ventro-lateral stridulatory ridge of abdominal segments IV–VI **K** stridulatory ridge **L** anal plate **M** peg-shaped hairs on hind tibia **N** marginal tubercle on prothorax **O** marginal tubercle on abdominal segment I **P** marginal tubercle on abdominal segment VII **Q** gonapophyses. Scale bars: 0.10 mm. (**A, N** from HL_20160627_3_A; **E, F, I, O** from HL_20150517_5_A; **C, G, J, K, M, P, Q** from HL_20150517_5_B; **B, D, H, L** from HL_20150517_5_C.).

***Thorax*.** Dorsal and ventral cuticle with polygon reticulations. Mesosternal furca with separated arms. Length of single arms 0.09–0.11 mm, 0.4–0.5 times as long as antennal segment III. Spiracles elliptical, spiracular plates dark brown. Prothorax with one pair of small marginal tubercles. Dorsal setae on thorax short and pointed, with small tuberculate bases. Legs normal. Distal part of femora, basal and distal part of tibiae dark brown, others brown. Hind femur 1.3–1.6 times as long as antennal segment III, hind tibia 0.5–0.6 times as long as body. Hind tibia with 7–8 peg-shaped spines, on basal two-thirds of inner side. Length of hairs on hind tibia 0.02–0.03 mm, 1.0–1.5 times as long as the widest diameter of antennal segment III. First tarsal chaetotaxy: 3, 3, 2. Second tarsal segments with transverse imbrications.

***Abdomen*.** Abdominal segments IV–VI with ventro-lateral spinulose ridges, forming a stridulatory surface. Marginal tubercles on abdominal segments I and VII. Abdominal dorsal hair sparse, fine, with tuberculate bases. Abdominal tergite VIII with two hairs. Siphunculi dark brown, cylindrical, with broad base, tapering towards the apex, with spinulose transverse imbrications, without flange or hairs. Siphunculi 0.12–0.17 mm, 1.9–2.8 times as long as its basal diameter, 0.9–1.2 times as long as cauda. Cauda short tongue-shaped, constricted in middle, 1.4–2.3 times as long as its basal diameter, with 14–21 hairs. Anal plate broad and round, with 18–21 hairs. Genital plate transversely oval, with 11–18 hairs. Cauda, anal plate and genital plate dark brown with dense spinules. Gonapophyses three, each with 3–5 hairs.

#### Specimens examined.

***Holotype***: apterous viviparous female, **China**: Fujian (Fuzhou, 26.1°N, 119.3°E, Alt. 258 m), 27 June 2016, No. HL_20160627_3_A, on *Adinandramillettii*, coll. X. L. Huang and X. L. Lin (FAFU). ***Paratypes***: 4 apterous viviparous females (No. HL_20150517_5_A, No. HL_20150517_5_B, No. HL_20150517_5_C and No. HL_20150517_5_D), **China**: Fujian (Fuzhou, 26.1°N, 119.3°E, Alt. 258 m), 17 May 2015, on *Adinandramillettii*, coll. X. L. Huang and X. L. Lin (FAFU).

#### Etymology.

The new species is named after FAFU, the abbreviation for Fujian Agriculture and Forestry University, where the samples of this species were first discovered and collected. And ‘fafuensis’ is an adjective of feminine gender in accord with the feminine *Aphis*.

#### Host plant.

*Adinandramillettii* (Hook. & Arn.) Benth. & Hook.f. ex Hance (Pentaphylacaceae).

#### Distribution.

China: Fujian Province (Fuzhou, Quanzhou and Wuyishan).

#### Biology.

This species feeds on shoots and undersides of young leaves of the host plant, and can be attended by at least two species of *Crematogaster* (Fig. 4B, C) according to our records.

#### Taxonomic notes.

Aphis (T.) fafuensis Cheng & Huang, sp. nov. has black-and-white banded antennae. Siphunculi and cauda are dark. Most part of femora, basal and distal parts of tibiae are dark brown. The peg-like spines on the hind tibiae and roughened ventro-lateral cuticle on the posterior part of the abdomen form a typical stridulatory apparatus. Compared with *A.aurantii*, the new species has a smaller body size and stubbier siphunculi: body length 0.91–1.19 mm (*A.aurantii*: 1.14–1.71 mm), siphunculi length 1.9–2.8 times of siphunculi basal width (*A.aurantii*: 2.0–3.8 times). The results of ANOVA analysis showed that there were significant differences between A. (T.) fafuensis Cheng & Huang, sp. nov. and A. (T.) sennae Cheng & Huang, sp. nov. and A. (T.) aurantii in some characters, such as the length of URS_BW, and the ratios of Ant. I and Ant. II to WA (Suppl. material 1: table S2).

### ﻿ Aphis (Toxoptera) sennae

Taxon classificationAnimaliaHemipteraAphididae

Cheng & Huang sp. nov.

627E4E24-D832-56B3-AF87-BECDCDD00C30

https://zoobank.org/3C5B676B-AEDE-4757-9136-AD087DB0E2D3

Figs 2 , 4D

#### Description.

Apterous viviparous females: Body pear-shaped, reddish brown in life, with black-and-white banded antennae and dark head, femurs, siphunculi and cauda (Fig. 4D).

**Mounted specimens: *Head*.** Dorsum of head smooth. Antennal tubercles slightly developed. Median frontal tubercle developed, slightly below antennal tubercles. Dorsal hairs 6–7, fine, with small developed tuberculate bases. Head with one pair of cephalic hairs, one pair of antennal tubercular hairs. Antennae six-segmented, segments I and II smooth, dark brown, segments III–VIb and PT imbricated, dark at distal end. Whole antennae 0.8–0.9 times as long as body. Length in proportion of segments I–VI: 19–25, 19–22, 100, 59–69, 61–69, 25–31 + 94–116. Processus terminalis 3.4–4.1 times as long as basal part of the segment. Antennal segments I–VI each with 4, 4, 5–9, 3–5, 3–4, 2–3 + 4–6 hairs, respectively, apex of processus terminalis usually with 3–4 hairs. Length of hairs on segment III 0.01 mm, 0.3 times as long as the widest diameter of segment III. Rostrum reaching hind coxae. Ultimate rostral segment wedge-shaped, 2.0–3.3 times as long as basal width, 1.2–1.4 times as long as second hind tarsal segment. Ultimate rostral segment with three pairs of hairs, including one pair of accessory hairs.

**Figure 2.**
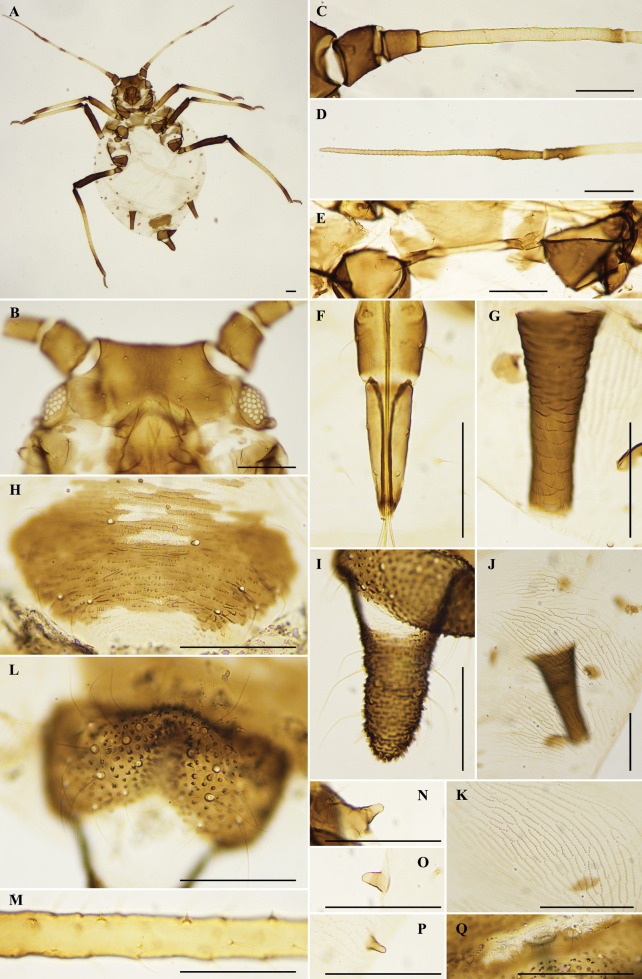
Aphis (Toxoptera) sennae Cheng & Huang, sp. nov., apterous viviparous female **A** dorsal view of body **B** dorsal view of head **C** antennal segments I–III **D** antennal segments V–VI **E** mesosternal furca **F** ultimate rostral segment **G** siphunculus **H** genital plate **I** cauda **J** ventro-lateral stridulatory ridge of abdominal segments IV–VI **K** stridulatory ridge **L** anal plate **M** peg-shaped hairs on hind tibia **N** marginal tubercle on prothorax **O** marginal tubercle on abdominal segment I **P** marginal tubercle on abdominal segment VII **Q** gonapophyses. Scale bars: 0.10 mm. (**D, J, Q** from HL_zld20171111_7_A; **B, H, M** from HL_zld20171111_7_B; **L** from HL_zld20171111_7_C; **A, E, G, I, K, N, O, P** from HL_zld20171111_7_D; **B** from HL_zld20171111_7_G; **F** from HL_zld20171111_7_H.).

***Thorax*.** Mesosternal furca with separated arms. Length of single arms 0.10–0.14 mm, 0.3–0.4 times as long as antennal segment III. Prothorax with one pair of small marginal tubercles. Dorsal hairs on thorax short and thin, with small tuberculate bases. Legs normal. Distal part of femora, basal and distal part of tibiae dark brown, others brown. Hind femur 1.4–1.5 times as long as antennal segment III. Hind tibia 0.5–0.6 times as long as body, with 8–10 peg-shaped spines, on basal two-thirds of inner side. Length of hairs on hind tibia 0.02–0.03 mm, 0.8–1.0 times as long as the widest diameter of antennal segment III. First tarsal chaetotaxy: 3, 3, 2. Second tarsal segments with transverse imbrications.

***Abdomen*.** Abdominal segments IV–VI with ventro-lateral spinulose ridges, forming a stridulatory surface. Abdominal segments I and VII each with one pair of marginal tubercles. Abdominal dorsal hair sparse, fine, with tuberculate bases. Abdominal tergite VIII with two hairs. Siphunculi dark brown, cylindrical, tapering towards the apex, with spinulose transverse imbrications, without flange or hairs. Siphunculi 0.17–0.20 mm, 1.9–2.7 times as long as its basal diameter, 0.9–1.1 times as long as cauda. Cauda short tongue-shaped, constricted in middle, 1.2–1.8 times as long as its basal diameter, with 9–17 hairs. Anal plate broad, with 20–29 hairs. Genital plate transversely oval, with 8–13 hairs. Cauda, anal plate and genital plate dark brown with dense spinules. Gonapophyses three, each with 4–5 hairs.

**Figure 3.**
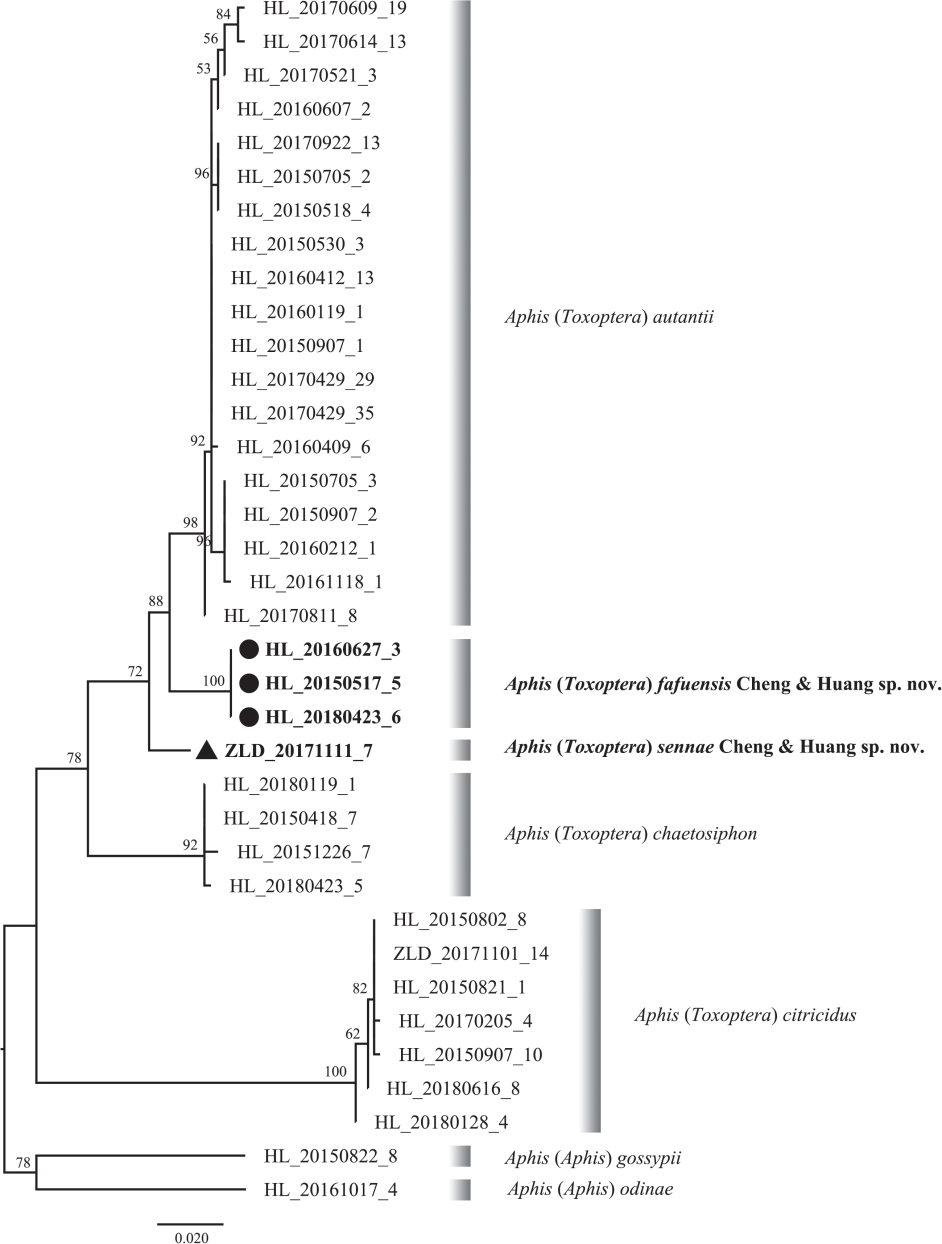
The maximum-likelihood phylogenetic tree of Aphis (Toxoptera) samples based on COI sequences. Numbers beside main nodes are bootstrap support values (>50). Solid circles and triangle mark the new species.

#### Specimens examined.

***Holotype***: apterous viviparous female, **China**: Yunnan (Kunming, 25.1°N, 102.7°E, Alt. 1900 m), 11 Nov. 2017, No. HL_zld20171111_7_A, on *Sennabicapsularis* coll. L. D. Zeng (FAFU). ***Paratypes***: 7 apterous viviparous females (No. HL_zld20171111_7_B, No. HL_zld20171111_7_C, No. HL_zld20171111_7_D, No. HL_zld20171111_7_E, No. HL_zld20171111_7_F, No. HL_zld20171111_7_G and No. HL_zld20171111_7_H), with the same collection date as holotype (FAFU).

#### Etymology.

The new species is named after the genus name of the host plant, *Sennabicapsularis*. The word ‘sennae’ is a noun, and does not change spelling based on gender.

#### Host plant.

*Sennabicapsularis* (L.) Roxb. (Fabaceae).

#### Distribution.

China: Yunnan Province (Kunming).

#### Biology.

It seems the species feeds on seed pods of the host plant.

#### Taxonomic notes.

Aphis (T.) sennae Cheng & Huang, sp. nov. has black-and-white banded antennae, and processus terminalis are dark, different from *A.aurantii* whose processus terminalis are dark basally and distally. Siphunculi length 1.9–2.7 times of siphunculi basal width (*A.aurantii*: 2.0–3.8 times). The body length of A. (T.) sennae Cheng & Huang, sp. nov. is 1.50–1.89 mm, which is significantly larger than A. (T.) fafuensis Cheng & Huang, sp. nov. (0.91–1.19 mm). Body color of A. (T.) sennae Cheng & Huang, sp. nov. is reddish brown, the head is slightly darker, and immatures are almost the same color as adult apterae. Adult apterae of *A.aurantii* and A. (T.) fafuensis Cheng & Huang, sp. nov. are brownish-black, the nymphs of these two species are lighter in body color, or reddish brown. The results of ANOVA analysis and the LSD test revealed significant differences between A. (T.) sennae Cheng & Huang, sp. nov. and A. (T.) fafuensis Cheng & Huang, sp. nov., as well as between A. (T.) sennae Cheng & Huang, sp. nov. and A. (T.) aurantii, in a number of characters, including the measured length, ratio, and number of hairs on various body parts (Suppl. material 1: table S2).

**Figure 4.**
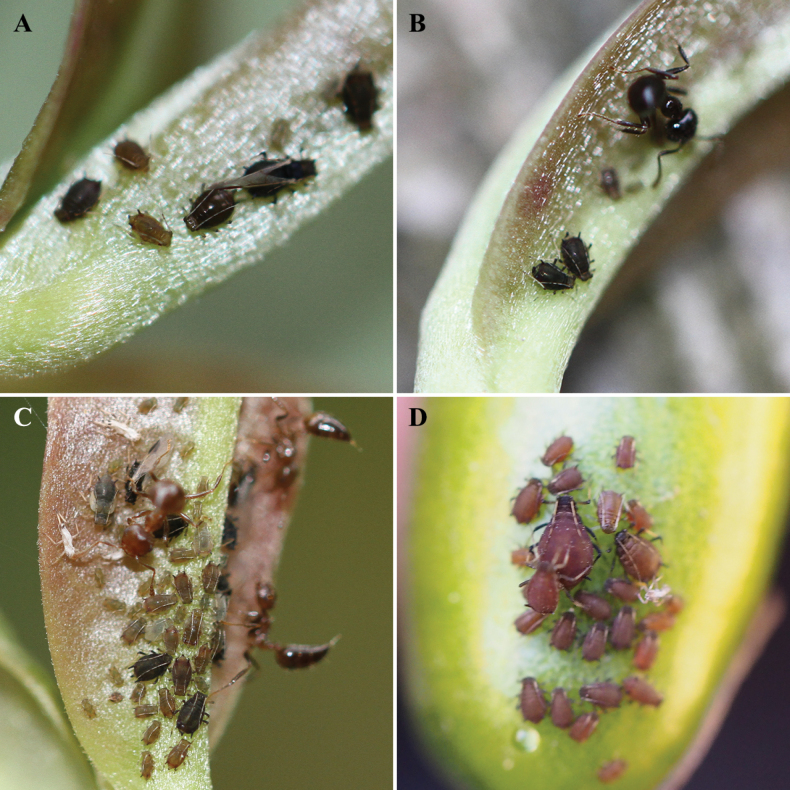
**A–C**Aphis (Toxoptera) fafuensis Cheng & Huang, sp. nov., colony on the shoot and the underside of leaf of *Adinandramillettii***D**Aphis (Toxoptera) sennae Cheng & Huang, sp. nov., colony on the seed pod of *Sennabicapsularis*.

##### ﻿Molecular analyses

The mean interspecific distance between A. (T.) fafuensis and A. (T.) aurantii was 2.8%, and the K2P distances between A. (T.) fafuensis and other species from Aphis (Toxoptera) ranged from 5.6% to 9.2%. Meanwhile, the mean interspecific distance between A. (T.) sennae and A. (T.) aurantii was 2.7%, and the K2P distances between A. (T.) sennae and other species within Aphis (Toxoptera) ranged from 4.5% to 8.2%. The averages of pairwise sequence divergences of the *COI* genes among thirty-six samples are presented in Table 3.

**Table 3.** Mean genetic distances (K2P) among two new species and three known species of subgenus Aphis (Toxoptera) based on COI sequences. The percentage of genetic distances are shown in the lower left half of the matrix, and the percentage of standard errors are shown in the upper right half of the matrix.

	A. (A.) gossypii	A. (A.) odinae	A. (T.) sennae Cheng & Huang, sp. nov.	A. (T.) fafuensis Cheng & Huang, sp. nov.	A. (T.) aurantii	A. (T.) chaetosiphon	A. (T.) citricidus
**A. (A.) gossypii**		1.24	1.02	1.14	1.17	1.10	1.42
**A. (A.) odinae**	8.18		1.25	1.19	1.24	1.13	1.36
**A. (T.) sennae Cheng & Huang, sp. nov.**	6.55	7.99		0.74	0.71	0.87	1.25
**A. (T.) fafuensis Cheng & Huang, sp. nov.**	7.35	7.59	2.94		0.69	0.98	1.37
**A. (T.) aurantii**	7.64	7.99	2.73	2.76		0.95	1.28
**A. (T.) chaetosiphon**	6.75	7.52	4.55	5.78	5.40		1.28
**A. (T.) citricidus**	10.38	9.24	7.95	9.05	8.32	8.77	

The phylogenetic results showed that A. (T.) fafuensis Cheng & Huang, sp. nov. and A. (T.) sennae Cheng & Huang, sp. nov. clustered together with the known species of Aphis (Toxoptera). Both the two new species showed morphologically and phylogenetically closer relationships with A. (T.) aurantii and A. (T.) fafuensis had a sister relation with A. (T.) aurantii, probably due to closer relationship of their host plants.

### ﻿Key to the species of Aphis (Toxoptera) species, apterous viviparous females

**Table:** 

1	Siphunculi usually 0.40–0.70 times as long as cauda in length	**2**
–	Siphunculi usually 0.90–1.90 times as long as cauda in length	**3**
2	Dorsal hairs of body with distinct sclerotizations at base; siphunculus with 6–8 hairs; cauda with 18–28 hairs; on *Camelliaoleifera*	**A. (T.) chaetosiphon**
–	Dorsal hairs of body without distinct sclerotizations at base; siphunculus without hairs or occasionally 1 hair; cauda with at most 22 hairs; on various plants	**A. (T.) victoriae**
3	Antennal segments III–IV pale, hairs on antennal segment III are the same length as the widest diameter of the segment or slightly longer; siphunculi with dense spinulose transverse imbrications over surface	**A. (T.) citricidus**
–	Apical part of antennal segments III and apical part of antennal segments IV dark, hairs on antennal segment III mostly 0.25–0.50 times as long as the widest diameter of the segment; siphunculi with spinulose transverse imbrications, but less distinct	**4**
4	Antennal segments III with 15–17 hairs; siphunculi usually 1.20–1.90 times as long as cauda in length	**A. (T.) aurantii**
–	Antennal segments III with at most 10 hairs, siphunculi usually 0.90–1.20 times as long as cauda in length	**5**
5	Apical part of processus terminalis dark, other parts of processus terminalis pale; body smaller, 0.90–1.20 mm in length; on *Adinandramillettii*	**A. (T.) fafuensis Cheng & Huang, sp. nov.**
–	Processus terminalis dark; body larger, 1.50–1.90 mm in length; on *Sennabicapsularis*	**A. (T.) sennae Cheng & Huang, sp. nov.**

## Supplementary Material

XML Treatment for Aphis (Toxoptera) fafuensis

XML Treatment for Aphis (Toxoptera) sennae

## ﻿Additional information

### Author ORCIDs

Zhentao Cheng https://orcid.org/0000-0003-3452-4075

Xiaolei Huang https://orcid.org/0000-0002-6839-9922

### Data availability

All of the data that support the findings of this study are available in the main text or Supplementary Information.

## Supplementary materials

Supplementary material 1 Results of one-way ANOVA analysis and post hoc LSD for morphological measurement Data type tables (Excel spreadsheet) Explanation note **table S1**: Detailed collection information for two new species, A. (T.) fafuensis Cheng & Huang, sp. nov. and A. (T.) sennae Cheng & Huang, sp. nov.; **table S2**: Results of one-way ANOVA analysis and post hoc LSD for morphological measurements of the A. (T.) fafuensis Cheng & Huang, sp. nov. (*N* = 6), A. (T.) sennae Cheng & Huang, sp. nov. (*N* = 8) and A. (T.) aurantii (*N* = 6). https://binary.pensoft.net/file/883238This dataset is made available under the Open Database License (http://opendatacommons.org/licenses/odbl/1.0/). The Open Database License (ODbL) is a license agreement intended to allow users to freely share, modify, and use this Dataset while maintaining this same freedom for others, provided that the original source and author(s) are credited.Zhentao Cheng, Xiaolei Huang
